# Macrophage heterogeneity in liver fibrosis

**DOI:** 10.3389/fimmu.2025.1639455

**Published:** 2025-09-04

**Authors:** Wenzhao Wang, Shengwen Li, Yanjun Liu, Xin Ding, Yongqi Yang, Shiyun Chen, Jiafan Cao, Frank Tacke, Wei Dong, Tian Lan

**Affiliations:** ^1^ Department of Pharmacology, College of Pharmacy, Harbin Medical University, State Key Laboratory of Frigid Zone Cardiovascular Diseases, Harbin, China; ^2^ School of Pharmacy, Guangdong Pharmaceutical University, Guangzhou, China; ^3^ Department of Pharmacy, The People’s Hospital of Miyi County, Panzhihua, China; ^4^ Department of Hepatology and Gastroenterology, Charité Universitätsmedizin Berlin, Campus Virchow Klinikum and Campus Charité Mitte, Berlin, Germany; ^5^ Department of Hepatopancreatobiliary Surgery, Harbin Medical University Cancer Hospital, Harbin, China

**Keywords:** macrophage, liver fibrosis, heterogeneity and plasticity, mechanisms, therapeutic approaches

## Abstract

Liver fibrosis represents a universal pathological endpoint in chronic hepatic disorders, in which hepatic macrophages play a pivotal role through dynamic phenotypic modulation. These versatile immune cells undergo functional and phenotypic transformations mediated by diverse molecular mediators, with their heterogeneity arising from both cellular origin differences and disease-specific microenvironments. The development of technologies such as single-cell and spatial omics has broken through the traditional M1/M2 classification paradigm of macrophages, revealing the molecular signatures and functional distinctions of hepatic macrophages during liver injury, fibrogenesis, and regression. Hepatic macrophages are central to the pathogenesis of chronic liver injury and considered as potential targets for drug discovery. While numerous macrophage-targeting strategies for liver fibrosis intervention currently remain in preclinical development, advancing our comprehension of macrophage plasticity and subset-specific functions holds significant potential. A deeper understanding of macrophage heterogeneity could provide a new therapeutic strategy against liver fibrosis, ultimately improving clinical outcomes for patients with chronic liver diseases.

## 1 Introduction

Liver fibrosis emerges as a common pathological consequence of chronic liver diseases. It is characterized by an excessive accumulation of extracellular matrix (ECM) proteins, largely derived from activated hepatic stellate cells (HSCs), culminating in the formation of fibrotic scar tissue (1). During this process, hepatic macrophages serve a critical function (2). Hepatic macrophages, including both Kupffer cells (KCs) and recruited macrophages, constitute a heterogeneous population of immune cells characterized by remarkable functional and molecular diversity (3). Their strategic positioning at the interface of hepatic blood flow and the sub-sinusoidal space of Disse, coupled with their heightened sensitivity to microenvironmental factors and high phagocytic capabilities, enable KCs to perform a variety of roles. These include immune responses, protection against infections, and the modulation of metabolic processes (4). This heterogeneity manifests through distinct cytokine profiles, surface marker expression patterns, and transcriptomic signatures, which collectively define their phenotypic identity. Macrophages are also extremely plastic, as demonstrated by their ability to alter their phenotype to adapt to the liver microenvironment and perform different functions (5). Injury-induced inflammation prompts the recruitment of macrophages to the liver, where they secrete pro-inflammatory cytokines that activate HSCs, thereby initiating liver fibrosis (6). In contrast, their phenotypic transition leads to the breakdown of extracellular matrix components and the secretion of cytokines with anti-inflammatory properties (7).

Hepatic macrophages play an important role in maintaining the dynamic balance of the liver and the pathogenesis of both acute and chronic liver injury. They are involved in various processes related to liver disease, such as exacerbating injury, reducing inflammation, promoting tissue repair, and influencing fibrosis progression and regression, as well as tumor promotion and suppression (8). These discoveries are catalyzing the development of macrophage-centric therapeutic strategies, with emerging evidence underscoring their potential for improving clinical management of chronic liver diseases. Therefore, we summarize therapeutic approaches that target hepatic macrophages for liver fibrosis. With the current improved understanding of the complex heterogeneity and functional diversity of macrophages, therapies targeting macrophages may represent a promising avenue for the treatment of liver fibrosis.

## 2 Origin of hepatic macrophages

### 2.1 Kupffer cells

KCs originate from yolk sac-derived colony-stimulating factor 1 receptor (CSF1R)^+^ erythromyeloid progenitors (EMPs) (9). Hepatic Transforming Growth Factor-beta (TGF-β) and desmosterol synergistically regulate SMAD and Liver X receptor (LXR) signaling pathways to maintain KCs identity (10). In healthy livers, KCs are mainly confined to the hepatic sinusoids and do not migrate, whereas monocyte-derived macrophages can be found extravascularly (11). KCs-specific markers in mice include C-type lectin domain family 4 member F (CLEC4F), V-set and immunoglobulin domain containing 4 (VSIG4), C-type lectin domain family 2 (CLEC2), and Folate receptor 2 (FOLR2), whereas in humans no consensus has been reached (12). KCs express a wide range of pattern recognition receptors (PRRs), including toll-like receptors (TLRs), nucleotide-binding oligomerization domain-like receptors (NLRs), and retinoic acid-inducible gene I-like receptors (RLRs) (13). KCs help maintain liver homeostasis and play important modulatory roles in bacterial clearance, antigen presentation, and modulation of iron/lipid metabolism (14).

### 2.2 Monocyte-derived macrophages

In the healthy liver, monocyte-derived macrophages (MoMϕs) predominantly localize to the portal triad region, where they maintain iron homeostasis and regulate cholesterol metabolism (15). MoMϕs are Cluster of Differentiation 11b (CD11b)^+^, F4/80^intermediate (int)^, Lymphocyte antigen 6 complex locus C (Ly6C)^+^ and CSF1R^+^, which are derived from bone marrow (BM) C-X3-C motif chemokine receptor 1 CX3CR1^+^ CD117^+^Lin^-^ progenitor cells (16, 17). These MoMϕs are primarily recruited to the liver by chemokines, such as C-C motif chemokine ligand 2 (CCL2), CCL1, and their receptors C-C chemokine receptor type 2 (CCR2) and CCR8 (18). The murine system features two principal circulating monocyte subsets characterized by Ly-6C expression levels: pro-inflammatory Ly-6C high (Ly-6C^hi^) monocytes and patrolling Ly-6C low (Ly-6C^low^) monocytes (19). In humans, monocytes are classified by their expression of CD14 and CD16 as classical (CD14^hi^CD16^−^), intermediate (CD14^+^CD16^+^) and non­classical (CD14^−^CD16^hi^) monocytes, which to some extent correspond to Ly-6C^hi^ and Ly-6C^low^ monocytes in mice respectively (20). Ly-6C^hi^ monocytes are characterized by their expression of inflammatory chemokine receptors, pattern recognition receptors, and cytokines, whereas Ly-6C^low^ monocytes demonstrate a patrolling function within the liver and exhibit a higher expression of scavenging receptors (20). Notably, phenotypic plasticity exists between these subsets. Ly-6C^hi^ MoMϕs can transition to a restorative Ly-6C^low^ phenotype through distinct mechanisms: phagocytic activity or exposure to interleukin-4 (IL-4) and IL-33 released by necrotic KCs (18). This phenotypic switching represents a critical adaptive mechanism in the process of hepatic fibrosis. Multiple lineage-tracing models have shown that MoMϕs are also the major population of immunosuppressive and liver metastasis-associated macrophages (LMAM) (21). Furthermore, MoMϕs can replace KCs when they are experimentally depleted due to liver injury, and these macrophages can subsequently acquire a phenotype that is almost identical to that of KCs (22, 23).

### 2.3 Peritoneal and splenic macrophages

Peritoneal macrophages (PMs), which are located in the peritoneal cavity, may migrate into the liver. PMs selectively express the transcription factor GATA6, which is not expressed by either liver-resident KCs or circulating monocytes (24). In the context of acute liver injury, silencing the pro-inflammatory protein High mobility group protein B1 (HMGB1) in liver-infiltrating PMs alleviates the liver injury phenotype in mice (25). However, it has been suggested that PMs do not deeply infiltrate the liver parenchyma during liver injury, which seems to contradict the conclusions of relevant studies (26, 27).

Splenic macrophages (SMs) exhibit regulatory roles in liver homeostasis and pathology. SMs express CD11b and CD115 but show low or no expression of CD90, B220, CD49b, NK1.1, and Ly-6G surface proteins (28). SMs enhance the secretion of CCL2 by hepatic macrophages, which in turn facilitates monocyte recruitment and the augmentation of liver fibrosis (29). In another study, a subtype of spleen‐derived monocytes identified as CD11b^+^CD43^hi^Ly6C^lo^ cells has been demonstrated to preferentially infiltrate fibrotic liver tissue and adopt macrophage characteristics, thereby exacerbating fibrogenesis (30). However, it remains controversial whether SMs can migrate to the liver. These hypotheses require more advanced imaging techniques or cell tracking methods to validate the migration pathways of PMs and SMs.

## 3 Heterogeneity and plasticity of macrophages

**Figure 1:**
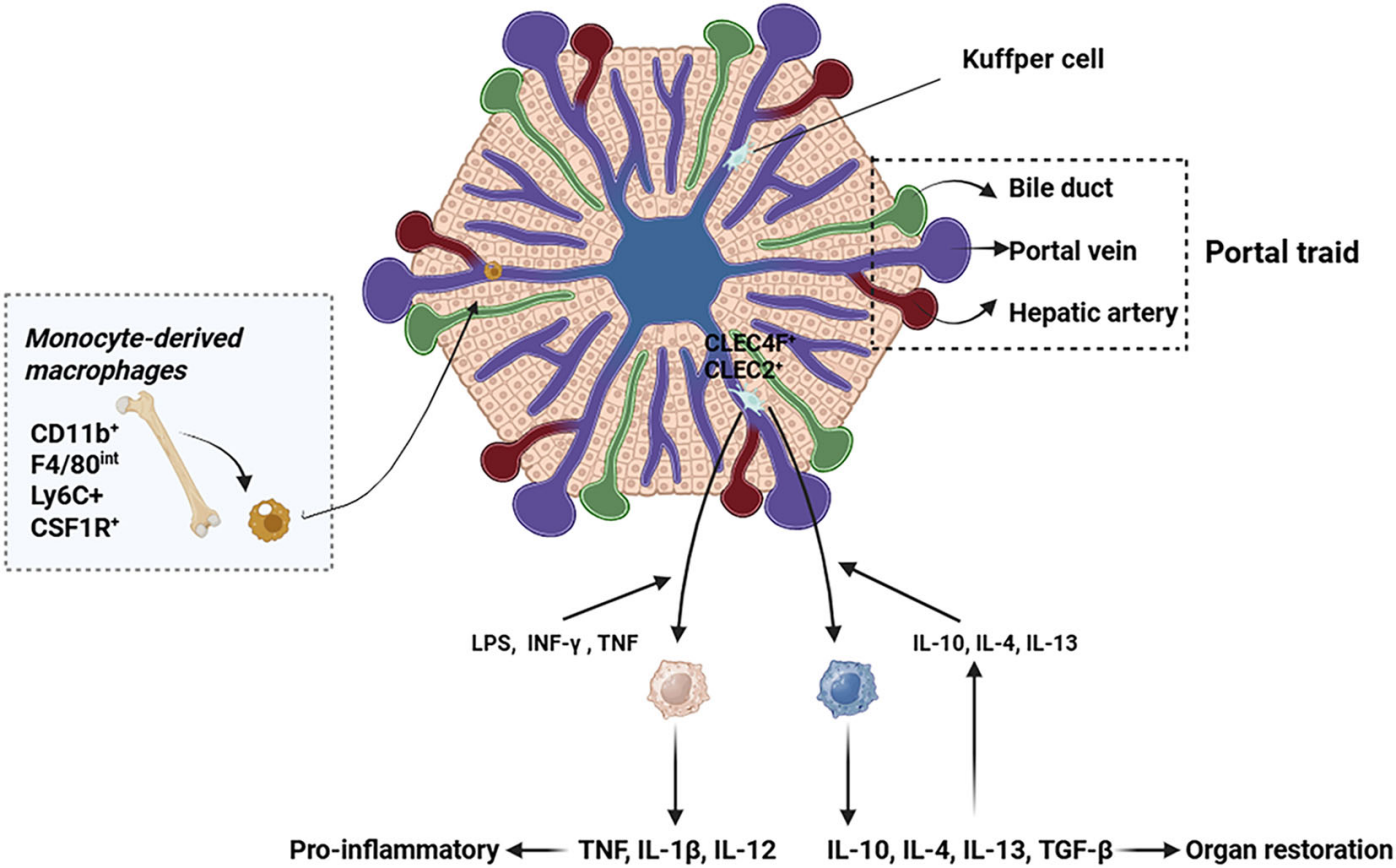
Origin and migration pathway of hepatic macrophages. This figure illustrates the heterogeneous origins and recruitment mechanisms of hepatic macrophages. KCs, the liver resident macrophages, are strategically positioned within the hepatic sinusoids. During liver injury, monocyte-derived macrophages are predominantly recruited to the liver via the systemic circulation.

The dynamic process of macrophage polarization entails the acquisition of specialized phenotypes and functional capabilities by macrophages as a reaction to stimuli present in their immediate surroundings. In 2000, Mills et al. categorized macrophages into two distinct subtypes, M1 and M2, based on differences in their metabolism, secretion, and function (31). This classification was based on the differential responses of macrophages *in vitro* to stimuli (32). Moreover, these polarized states demonstrate bidirectional interconversion when exposed to specific microenvironmental stimuli (33). Pro-inflammatory macrophages are typically triggered by stimulation with lipopolysaccharide (LPS), interferon-γ (IFN-γ), tumor necrosis factor (TNF), granulocyte-macrophage colony-stimulating factor (GM-CSF), and TLR ligands (34). Normally, pro-inflammatory macrophages are characterized by their robust secretion of pro-inflammatory cytokines, including TNF-α, interleukin-1 beta (IL-1β), and IL-12. These cytokines eventually drive the activation of HSCs and promote liver fibrosis progression (35). Additionally, pro-inflammatory macrophages generate substantial amounts of reactive oxygen species (ROS) and reactive nitrogen species (RNS), which collectively enable them to effectively kill invading pathogens, as well as phagocytose and clear senescent, damaged, and degenerated cells (36). In contrast, alternatively activated macrophages play a crucial role in defending against parasitic infections, participating in tissue remodeling and secreting immunomodulatory mediators such as IL-10, TGF-β, IL-4 and IL-13 (37). Among these cytokines, TGF-β plays a crucial role in HSCs activation and liver fibrosis (38) (**Figure 1**).

Recent studies have shown that the traditional M1/M2 paradigm for classifying macrophages has been rendered obsolete by new technological breakthroughs, particularly in characterizing the complexity of hepatic macrophage populations (39). Hepatic macrophages are heterogeneous in the healthy liver, comprising distinct subsets with unique transcriptional profiles and, consequently, distinct functional roles (40). While the traditional M1/M2 classification remains useful for a broad understanding, it is insufficient to capture the full spectrum of macrophage functionality. Instead, distinct macrophage subpopulations exhibit unique biological characteristics across various disease contexts, and the functional differences among these subpopulations play crucial roles in disease progression and treatment response. Moreover, reliance on this general M1/M2 classification may impede the development of targeted therapies tailored to specific diseases (31). Therefore, despite its utility as a foundational framework, the M1/M2 paradigm’s limitations in explaining and treating complex diseases have prompted researchers to adopt more refined macrophage subpopulation analyses. This nuanced approach facilitates the identification of specific roles for different macrophage subtypes in various diseases, providing an essential foundation for the development of personalized targeted therapies.

## 4 Mechanisms of macrophage polarization

**Figure 2:**
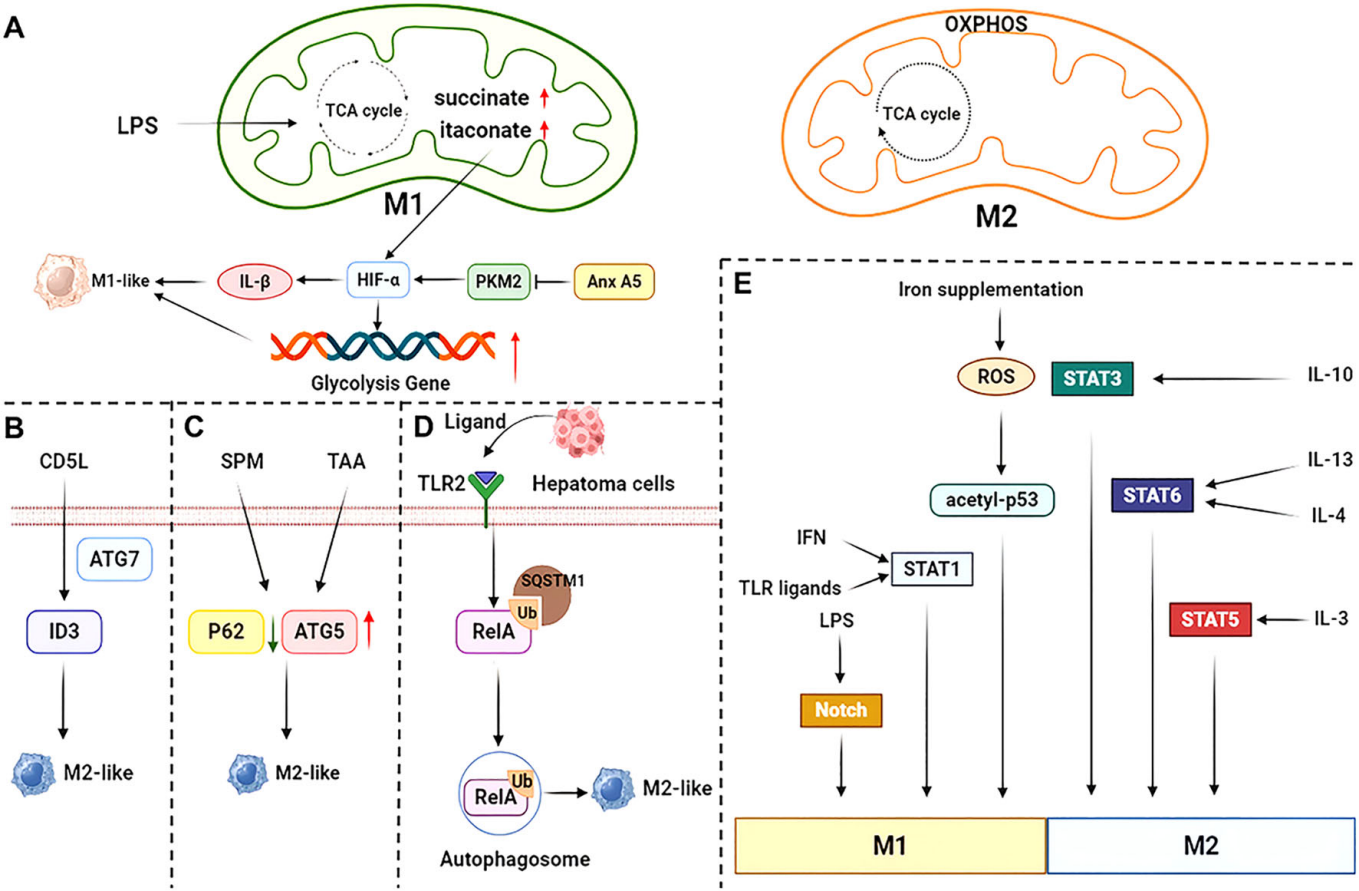
Mechanisms of macrophage polarization. **(A)** The tricarboxylic acid cycle is impaired in pro-inflammatory-polarized macrophages. Both Annexin A5 (Anx A5) and succinate upregulate HIF-α, which in turn promotes glycolytic gene expression and pro-inflammatory factor IL-1β expression. **(B)** CD5L induces ID3 expression and promotes alternatively activated polarization via the autophagy protein ATG7. **(C)** SPM-mediated KCs autophagy promotes alternatively activated polarization in TAA-induced liver injury through downregulation of P62 and upregulation of ATG5 expression. **(D)** Autophagy in hepatocellular carcinoma tissue is triggered by TLR2 ligand activation, which causes NF-κB RelA to be ubiquitinated and recognized by Sequestosome 1 (SQSTM1). This results in inhibition of the NF-κB pathway and consequently promotes alternatively activated polarization. **(E)** LPS, IFN, TLR ligands and iron supplementation promote pro-inflammatory polarization through different signaling pathways, while IL-10, IL-4, IL-13 and IL-3 promote alternatively activated polarization. SPM, spermine; TAA, thioacetamide.

The dynamic regulation of immune cell responses by environmental stimuli manifests particularly through modifiable macrophage activity and functional plasticity. This adaptive “short-term memory” mechanism induces transient yet sustained modifications in macrophage phenotypes, thereby dynamically influencing their pathogenic contributions during disease progression (41). Macrophage polarization is controlled by a variety of molecular mechanisms, mainly including metabolic reprogramming, autophagy, iron metabolism, Signal Transducer and Activator of Transcription (STAT) and Notch signaling pathways (35, 42) (**Figure 2**).

### 4.1 Metabolic reprogramming

Metabolic adaptations play a pivotal role in macrophage activation and fibrogenic processes (35). In quiescent conditions, macrophages predominantly utilize tricarboxylic acid (TCA) cycle coupled with oxidative phosphorylation (OXPHOS) to generate Adenosine triphosphate (ATP), establishing energy equilibrium through mitochondrial respiration (43). During macrophage polarization toward pro-inflammatory phenotypes, glycolysis is the predominant metabolic pathway, while the TCA cycle is disrupted at two key points. These interruptions lead to the accumulation of itaconate and succinate, which are critical metabolites that contribute to the pro-inflammatory phenotype of macrophages (44). Excessive succinate stabilizes hypoxia inducible factor-1α (HIF-1α), which in turn activates the transcription of glycolytic genes, thereby maintaining glycolytic metabolism in pro-inflammatory macrophages (44). Moreover, HIF-1α exerts a regulatory influence on sphingosine 1-phosphate (S1P) metabolism, thereby modulating the migration, activation, differentiation, and polarization of macrophages (45). However, recent research suggests that the stabilization of HIF-1α takes place at a later stage in the process of inflammatory macrophage polarization. Furthermore, it indicates that the initial production of lactate through glycolysis is not governed by HIF-1α (46). Pro-inflammatory macrophages are distinguished by their enhanced glycolysis, elevated levels of glutathione, increased expression of ferritin, upregulated expression of cyclooxygenase (COX) 2, low expression of COX1, robust activity of inducible nitric oxide synthase (iNOS), and diminished activity of arginase 1 (Arg1) (47).

In contrast, alternatively activated macrophages exhibit a greater dependence on OXPHOS. Their TCA cycle remains intact, providing essential substrates for the electron transport chain (ETC). The coupling of mitochondrial OXPHOS with the TCA cycle is a slower process but generates significantly more ATP through the ETC (48). Although glycolysis produces less ATP compared to OXPHOS, its rapid rate of ATP generation is crucial for maintaining energy levels, especially under conditions demanding a rapid response (49). These macrophages are characterized by augmented fatty acid oxidation (FAO), reduced expression of ferritin, lower levels of glutathione, decreased production of COX2, heightened COX1 expression, weak iNOS activity, and enhanced Arg1 activity (47). Among the myriad metabolic alterations, the divergent metabolism of L-arginine represents one of the earliest described and most distinctive features used to differentiate between pro-inflammatory and alternatively activated macrophages. iNOS and Arg1 serve as quintessential effector molecules for pro-inflammatory and alternatively activated macrophages, respectively (50).

Pyruvate kinase M2 (PKM2) is a key determinant of macrophage glycolytic reprogramming and maintenance of pro-inflammatory polarization (51). Follistatin-like protein 1 (FSTL1) binds directly to PKM2 and promotes PKM2 phosphorylation and nuclear translocation (52). Conversely, Annexin A5 targeting to PKM2 causes glycolysis inhibition and activation of mitochondrial oxidative metabolism, thereby triggering macrophages to switch to an anti-inflammatory phenotype (53). In addition, growth differentiation factor 15 (GDF15) reprograms macrophage metabolic pathways, leading them to acquire an OXPHOS-dependent anti-inflammatory functional fate (54). Collectively, these distinct metabolic adaptations are not merely energetic adaptations, but constitute essential regulatory nodes that biochemically enforce macrophage polarization while dynamically coordinating immune functionality within specific microenvironmental niches.

### 4.2 Autophagy

Autophagy is essential for maintaining cellular homeostasis and significantly contributes to macrophage development, while also influencing their apoptosis via modulation of colony-stimulating factors (55, 56). Previous studies showed that cell division cycle 5-like (CD5L) regulates the up-regulation of inhibitor of DNA binding 3 (ID3) through the autophagy-related gene 7 (ATG7) and promotes an anti-inflammatory cytokine profile in response to TLR activation (57). In Thioacetamide (TAA)-induced KCs injury, spermine (SPM) pretreatment decreases P62 protein expression and increases ATG5 protein expression, thereby promoting anti-inflammatory polarization (58). Furthermore, TLR2-induced autophagosomal degradation of NF-κB RelA (P65) inhibits the NF-κB signaling pathway and drives alternatively activated macrophage polarization (59). In addition, it has been demonstrated that enhancing macrophage autophagy flux through ubiquitin-specific protease 19 (USP19) promotes the polarization of macrophages towards an anti-inflammatory phenotype (60). Fibroblast growth factor 21 (FGF21) significantly attenuates pro-inflammatory macrophage activation through autophagy-mediated degradation of HIF-1α (61). Additionally, inhibition of macrophage autophagy promotes M2-like polarization through ubiquitination-mediated degradation of TGF-β-activated kinase 1 and MAP3K7-binding protein 3 (TAB3), resulting in destabilization of the NF-κB signaling pathway (62). Collectively, these findings underscore the multifaceted role of autophagy in modulating macrophage polarization.

### 4.3 Iron metabolism

Iron homeostasis and the expression of iron-related genes strikingly shift during macrophage polarization, indicating a potential role for iron in macrophage activation. For example, in pro-inflammatory macrophages, the expression of Hepcidin antimicrobial peptide (Hamp) and FtH/FtL is highly upregulated, while Ferroportin (FPN) and IRP1/2 are downregulated (63). Upregulation of iron uptake and storage activates liver macrophages through the NF-κB pathway (64). Iron overload can polarize macrophages to the pro-inflammatory phenotype through the ROS/acetyl-p53 pathway (65). A recent study has shown that glycyrrhetic acid 3-O-mono-β-d-glucuronide (GAMG) induces ferroptosis of inflammatory macrophages through downregulation of solute carrier family 7 member 11 (SLC7A11) (66). However, exogenous iron supplementation and iron-rich ECM from human dermal fibroblasts induce the polarization of THP-1 cells and bone marrow-derived macrophages (BMDMs) into alternatively activated macrophages (67). These studies demonstrate the complexity of iron metabolism in macrophage polarization and function.

### 4.4 STAT signaling pathway

The STAT signaling pathway is a crucial mediator of cytokine signaling (e.g., IL-4, IL-6, IFN-γ) (68). Its core mechanism involves ligand binding to transmembrane receptors, which triggers JAK phosphorylation and subsequent STAT protein activation. Phosphorylated STAT proteins dimerize, translocate to the nucleus, and regulate target gene expression (69). In macrophage polarization, this pathway modulates the transition between pro-inflammatory and anti-inflammatory phenotypes through selective activation of distinct STAT isoforms (68). Specifically, IFN-γ and TLR-activated IRF-STAT1-signaling pathways orient macrophage function toward the pro-inflammatory phenotype, whereas IL-4 and IL-13 activate alternatively activated macrophages through STAT6 (70). Additionally, IL-10 and IL-3 activate STAT3 and STAT5, respectively, to promote alternatively activated macrophage polarization (71).

### 4.5 Notch signaling pathway

Notch signaling, known for its critical role in liver development, is also involved in liver regeneration, carcinogenesis, and metabolism (72). Macrophages express Notch ligands and receptors, indicating that Notch signaling participates in macrophage activation (73). LPS can upregulate Notch1 expression in macrophages via MyD88-dependent pathways, thereby induces the expression of its downstream genes (74). The Notch1 signaling pathway enhances the pro-inflammatory activation of hepatic macrophages by directly increasing the transcription of pro-inflammatory genes and by altering mitochondrial metabolism toward glucose oxidation, which leads to the production of mitochondrial reactive oxygen species (mtROS), further boosting the expression of pro-inflammatory genes (75).

## 5 The role of macrophages in liver fibrosis

Increasing evidence has shown that liver-resident macrophages and recruited monocyte-derived macrophages, which play an important role in liver fibrosis, are involved from initial liver injury and fibrosis formation to fibrosis regression (8). Among these cells, the existence of specialized subpopulations with distinct functional roles in health and disease has been documented (76).

Scar-associated macrophages (SAMs), which derive from BMDMs, accumulate in mouse fibrotic livers (77). There is a notable proliferation of the Scar-associated TREM2^+^ CD9^+^ macrophage subset. These cells are derived from circulating monocytes and play a role in enhancing the fibrotic response (78). In the initial phases of hepatic fibrosis, the activation of HSCs by macrophages through the release of inflammatory cytokines constitutes a pivotal mechanism that intensifies fibrosis (79). Macrophages accelerate fibrosis by secreting various cytokines, including TGF-β1, Vascular Endothelial Growth Factor (VEGF), and angiotensin II, which activate local tissue cells such as HSCs and myofibroblasts (80). Interestingly, activated HSCs further promote the transformation of macrophages into pro-inflammatory and pro-fibrogenic phenotypes. Activated HSCs attract monocytes/macrophages through the production of chemokines such as CCL2 and the infiltrating monocytes or macrophages can then further activate HSCs (81). For instance, sphingosine kinase 1 (SphK1) in KCs mediates CCL2 secretion, while SphK1 in HSCs upregulates CCR2 by downregulating miR-19b-3p (82). Furthermore, SphK1 aggravates liver fibrosis by promoting macrophage recruitment and M1/M2 polarization (83). The interaction between Jagged-1 on liver macrophages and Notch1 on HSCs drives Notch1-mediated HSCs activation and liver fibrosis (84). Additionally, MyD88 signaling in HSCs increases the secretion of CXCL10, which promotes macrophage polarization toward a pro-inflammatory phenotype and subsequent fibrosis (85).

Notably, in a carbon Tetrachloride (CCl_4_)-induced liver fibrosis model, macrophages exhibit contrasting functions in development and resolution of fibrosis: their elimination curbs development of fibrosis, while their absence following the cessation of injury hinders the resolution process, thereby worsening the fibrosis (86). In recent studies, the Ly6C^lo^CD11B^hi^F4/80^int^ macrophage population aggregates in the liver and constitute the main matrix metalloproteinase (MMP)-expressing macrophage subset during maximal fibrosis regression. It is crucial for degradation of tissue scar and originates from the infiltration of Ly-6C^hi^ inflammatory monocytes (87). Collectively, these observations highlight that the dual regulation between macrophages and HSCs is a principal driver of fibrosis advancement.

## 6 The role of macrophages in different liver fibrosis induced by multiple disease

### 6.1 Metabolic dysfunction-associated steatotic liver disease

Metabolic dysfunction-associated steatotic liver disease (MASLD), formerly known as nonalcoholic fatty liver disease, is characterized by excessive hepatic lipid accumulation (88). The spectrum of MASLD extends from hepatic steatosis to metabolic dysfunction-associated steatohepatitis (MASH), which may progress to advanced liver fibrosis, cirrhosis, or even hepatocellular carcinoma (HCC) (89). Recent single-cell sequencing analyses have revealed a significant characteristic of both human and murine MASH, namely the formation of crown-like macrophage clusters. These clusters are observed encircling hepatocytes that are either dead or dying, which are characterized by substantial lipid accumulation. Additionally, these macrophage aggregates are found in close proximity to regions exhibiting fibrosis and to areas where HSCs have been activated (39, 40). Macrophages are important mediators of the inflammatory response that underlies the progression of MASLD to fibrosis.

Under normal physiological conditions, KCs are attached to the space of Disse within the hepatic sinusoids, and display thin, silk-like or flat, plate-like pseudopodia. In contrast, during the course of steatohepatitis, KCs tend to form clusters and lose their typical villus-like or digit-like extensions (90). Knockdown of Jun N-terminal kinase-1/2 (JNK-1/2) in KCs reverses liver fibrosis in choline-deficient, L-amino acid-defined (CDAA) diet-fed mice and reduces inflammatory responses (91). Dietary fat and cholesterol can suppress type 1 cytokine expression and oppositely upregulate the type 2 cytokines in murine KCs (92). The equilibrium among macrophage polarization states significantly influences the advancement of steatohepatitis. For instance, arginase-2 knockout mice develop spontaneous steatohepatitis, which can be mitigated by KCs depletion (93). Histidine-rich glycoprotein (HRG), produced by hepatocytes, induces macrophage pro-inflammatory polarization, whereas HRG knockout mice are protected from experimental steatohepatitis (94).

As MASLD progresses, resident KCs in the liver are gradually replaced by recruited macrophages (95). KCs expressing TREM2 localize to sites of inflammation, hepatic damage and fibrosis, and soluble TREM2 correlates with disease severity in humans (96). Additionally, TREM2 ligation inhibits TLR4-driven inflammation in KCs (97). Single-cell analysis has revealed that recruited macrophages exist in two subsets with distinct activation states, either CCR2^+^, CX3CR1^+^, Ly-6C^hi^ monocytes or TREM2^+,^CD63^+^, CD9^+^ lipid-associated macrophages (LAMs) (98, 99). Genetic deletion of TREM2 in LAMs significantly impairs their tissue repair capacity, leading to exacerbated macrophage-mediated hepatic inflammation and accelerated fibrogenesis (100, 101). Given the crucial role of TREM2 in regulating both lipid metabolism and immune responses, therapeutic interventions targeting TREM2 modulation may offer promising novel strategies for the treatment of MASH (102). Another study has shown that hepatic LAMs express osteopontin (SPP1), a biomarker for patients with MASH, which is linked with the development of fibrosis (95). SPP1 has been reported to be upregulated in liver fibrosis and is tightly linked to dismal prognosis in end-stage hepatocellular carcinoma (103, 104). Studies have reported that myeloid-specific Glycoprotein Non-Metastatic Melanoma Protein B (GPNMB) knockout contributes to monocyte-derived macrophages occupation of the KCs niche and inhibits the formation of LAMs, thereby decreasing liver fibrosis (105). Another study suggested that the absence of Breast Regression Protein 39 (BRP39) reduces infiltration of LAMs, quelling liver inflammation and fibrosis (106). Notably, TREM2 also promotes lung fibrosis via protecting against macrophage apoptosis (107), while itaconate secreted by TREM2^+^ macrophages prevents apoptosis in cardiomyocytes and stimulates the growth of fibroblasts, which in turn enhances the process of cardiac tissue repair (108). Therefore, searching for new targets for LAMs is of great significance in the treatment of liver fibrosis in MASLD.

The genes elevated in MASLD have also been found to regulate macrophage polarization. In human and murine MASH, upregulated CD47 on necroptotic hepatocytes (necHC) and SIRPα on liver macrophages impair necHC uptake by liver macrophages, thereby promoting HSCs activation and fibrosis (109). Furthermore, macrophage-derived FGF12 and Tim3 have been shown to differentially activate HSCs through distinct mechanisms via the Monocyte Chemoattractant Protein-1 (MCP-1)/CCR2 axis and TGF-β secretion, respectively, all of which contribute to MASH pathogenesis (110, 111). Another study found that Niemann-Pick C1 (NPC1)-deficient macrophages exhibited inefficient efferocytosis in MASLD (112). HIF-1α, particularly in macrophages is increased in mice and patients with MASH, stimulating the release of inflammatory cytokines, which exacerbates both hepatic steatosis and inflammation (113). Simultaneously, it has been reported that macrophage HIF-2α mitigates insulin resistance and inflammation in adipose tissue by promoting an alternative activation polarization state (114). While PPARγ, rather than PPARδ, is essential for initiating the metabolic shift in response to IL-4, the deletion of either isoform has been demonstrated to hinder IL-4-triggered alternative macrophage activation, leading to insulin resistance and the development of hepatic steatosis (115, 116). Notably, a recent study identified a dopamine receptor D2 (DRD2) antagonist that selectively inhibits Yes-associated protein (YAP) in macrophages but not hepatocytes and thereby blocks the crosstalk between macrophages and the CTGF^+^VCAM1^+^ vascular niche, thereby promoting liver regeneration rather than fibrosis (117).

### 6.2 Alcoholic liver disease

Alcohol-associated liver disease (ALD) ranks among the most common liver conditions globally (118). Pericellular and perisinusoidal matrix accumulation with a chicken-wire appearance are also a characteristic fibrotic pattern in ALD (119). Alcohol consumption leads to malondialdehyde-acetaldehyde (MAA) adduct accumulation and stimulates KCs to produce IL-6, thereby accelerating hepatic inflammation and fibrosis in aldehyde dehydrogenase 2 (ALDH2) knockout mice (120). During ALD, the death of hepatocytes releases damage-associated molecular patterns, which in combination with necrotic cellular remnants and acetaldehyde—a byproduct of ethanol metabolism—induce the activation of KCs. This activation initiates hepatic inflammation through both innate and adaptive immune reactions (119, 121). Additionally, KCs produce nitric oxide (NO) and nicotinamide adenine dinucleotide phosphate (NADPH) oxidase, which further contribute to ALD (122).

Intestinal barrier dysfunction is an important contributor to ALD. Excessive alcohol consumption disrupts gut epithelial tight junctions, which increases intestinal permeability and facilitates the translocation of gut-derived LPS to the liver (123). During alcohol ingestion, high miR-212 expression suppresses zonula occludens-1 (ZO-1), a major component of tight junctions, causing disruption of gut integrity and permeability, thereby leading to LPS transport to the liver and subsequent activation of KCs (122, 124). Both KCs and activated HSCs contribute to fibrosis progression in alcohol-induced fibrosis through TLR4 (125). Silvia Affò et al. suggested that the upregulation of CCL20, mainly produced by macrophages, was strongly associated with LPS and silencing of CCL20 in mice reduces the expression of LPS-induced hepatic pro-inflammatory and pro-fibrogenic genes (126). In another study, monocyte-derived macrophages exhibit a pronounced inflammatory phenotype in a Notch-dependent manner (127).

### 6.3 Viral hepatitis

The global prevalence of viral hepatitis is predominantly attributed to five distinct hepatotropic viruses that are biologically unrelated, including hepatitis B virus (HBV), hepatitis C virus (HCV) among others (128). The estimated number of deaths due to viral hepatitis increased from 1.1 million in 2019 to 1.3 million in 2022, with 83% of deaths caused by HBV and 17% caused by HCV globally (129). Both adaptive and innate immunity are involved in the immune response to viral hepatitis, and the essential role of non-specific defense—especially the function of hepatic macrophages—has received wide attention (130).

A high HBV/HCV titer not only suppresses the polarization of pro-inflammatory macrophages, but also encourages their differentiation into a tolerogenic state (131). During immune activation in human and rodent infections, hepatitis B virus suppresses NF-κB pathway and ROS production in LPS-induced KCs, thereby inhibiting NOD-like receptor thermal protein domain associated protein 3 (NLRP3) inflammasome activation and IL-1β production (132). In agreement with the finding, HCV core protein can inhibit the NF-κB pathway to greatly reduce the expression of CCL2 and CXCL10 in macrophages (133). Similarly, HBV splicing-generated protein (HBSP) impacts liver monocyte/macrophage recruitment through a down-regulation of hepatocyte CCL2 expression upon acute liver injury (134).

In human liver, primarily via stimulating macrophages, IFN-λ not only drives antiviral responses, but also promotes inflammation and fibrosis in viral diseases (135). Research corresponding to this statement has uncovered that IFN-λ3, but not IFN-λ4, is likely to be the major IFN-λ subclass mediating hepatic inflammation and fibrosis progression in HCV patients (136). However, exposure of human naive liver macrophages to HBV leads to an increased proportion of anti-inflammatory macrophages, which favors HBV development by releasing IL-10 (137). Moreover, HBV stimulates monocyte/macrophage secretion of TGF-β (138), while inhibiting the secretion of IL-12 induced by TLR2 to induce immune suppression (139). A recent study has confirmed that TLR2 is the direct binding receptor of hepatitis B e-antigen (HBeAg), which promotes the proliferation of HSCs in a macrophage-dependent manner (140). Consistently, prokineticin 2 (PK2), as a potential cytokine expressed in KCs, modulates the number of pro-inflammatory cells, thereby regulating their role in the progression of liver fibrosis after HBV infection (141). In addition, the activation of Stimulator of Interferon Genes (STING) signaling suppresses macrophage inflammasome activation by activating autophagic flux to alleviate HBV-induced liver fibrosis (142). The present investigation identifies MMP9^+^ macrophages as the pivotal drivers of end-stage hepatocellular carcinoma in patients with chronic HCV infection (143).

### 6.4 Cholestatic disease

Cholestatic diseases such as primary biliary cholangitis (PBC) and primary sclerosing cholangitis (PSC) are characterized by the retention of bilirubin and bile salts in the liver and elevations of these metabolites in systemic circulation with a significant impact on organ function (144). The activation and recruitment of macrophages are mediated by ductular reactive cells (the epithelial cells characterized as a biliary phenotype) via the secretion of various factors (145). Exosomal lncRNA H19 derived from cholangiocytes enhances the pro-inflammatory polarization of KCs and promoted the recruitment and differentiation of BMDMs via inducing the expression and secretion of CCL2 and IL-6 in KCs (146). Flow cytometry analysis of non-parenchymal liver cells in PBC reveals massive infiltration of BMDMs in the liver, whereas the number of KCs decreases. These BMDMs exhibit high levels of TREM2 and SPP1 expression, which are characteristics of hepatic bile duct-associated macrophages. They are predominantly found surrounding the portal triad, a pattern that has been validated in patients with PSC (147). In contrast, SPP1^+^ macrophage infiltration in intrahepatic cholangiocarcinoma is associated with reduced tumor aggressiveness and improved patient survival (148). Another study has shown that high expression levels of IL-23 mRNA in CX3CR1^hi^CD11c^+^ BMDMs, inducing a significant intrahepatic increase in the frequency of hepatic IL-17A-producing CD4^+^ T cells and activity of the IL-23-IL17 axis, thereby aggravating PBC (149).

Moreover, KCs isolated from PBC mice showed increased surface RAE-1 protein expression and cytokine secretion, which subsequently activated NK cell-mediated target cell killing via Natural Killer Group 2 Member D (NKG2D)/Retinoic Acid Early Transcript 1 (RAE-1) recognition, increased inflammation, and fibrosis (150). IFN-γ further increased frequencies of inflammatory macrophages in the liver and aggravated liver fibrosis (151). In the absence of Protein Tyrosine Phosphatase 1B (PTP1B), which normally restricts the duration of pro-inflammatory signaling cascades, the activation and recruitment of hepatic macrophages are markedly enhanced after bile-duct ligation (BDL) (152). Macrophage phagocytosis of apoptotic cells was delayed by the induced high expression of CD16 in PBC BMDMs, promoting inflammation and fibrosis (153). In Mdr2^-/-^ mice, CCL24-driven macrophages induce proliferation of HSCs and cholangiocytes to promote cholestasis and fibrosis (154).

## 7 Therapeutic approach for targeting macrophage in liver fibrosis

Hepatic macrophages, including KCs and other resident macrophages, play a crucial role in maintaining liver homeostasis and modulating the progression or regression of liver fibrosis. These cells are of significant therapeutic interest due to their central role in normal tissue homeostasis and their dual functions in promoting and inhibiting fibrosis. As the first line of defense against liver injury, hepatic macrophages orchestrate both pro-fibrotic and anti-fibrotic responses, making them attractive targets for therapeutic intervention. Although most macrophage-based therapies have been tested primarily in experimental animal models, some have been evaluated in clinical trials (155). Emerging translational strategies focus on multidimensional modulation of macrophage biology:

- Dampening KCs activation: Targeting the activation of KCs to reduce pro-inflammatory signaling and subsequent fibrogenesis.
- Inhibiting the recruitment of inflammatory cells (monocytes and macrophages) to the injured liver: Preventing the recruitment of inflammatory cells, such as monocytes and macrophages, to the injured liver to mitigate excessive inflammation.
- Shaping the heterogeneity of liver macrophages: Shaping the diverse phenotypes and functions of hepatic macrophages to promote an anti-fibrotic environment.
- Augmenting the differentiation into restorative macrophages: Shifting the hepatic microenvironment from inflammation toward resolution, as well as enhancing restorative differentiation pathways in macrophages by delivering phagocytic stimuli.
- Cell-based therapies involving autologous macrophages infusion: Utilizing autologous macrophage infusion to introduce macrophages with specific anti-inflammatory or pro-resolving properties.
- Targeting macrophages with nanostructures: Employing nanostructures to selectively target and modulate macrophage function in the liver.

### 7.1 Dampening KCs activation

Emricasan, a pan-caspases inhibitor, reduces inflammation and apoptosis by inhibiting KCs activation caused by NLRP3 inflammasome cascades (156, 157). However, it did not improve liver fibrosis in patients with MASH (158).

Apoptosis signal-regulating kinase 1 (ASK1) is a ubiquitously expressed redox-sensitive regulator of both JNK and p38-mediated inflammation and apoptosis (159). KCs are activated by p38 and JNK in liver fibrosis and blocking the inflammatory signaling pathway of KCs can reduce inflammation and fibrosis in NASH. Selonsertib, an ASK1 inhibitor, failed to demonstrate improvement in liver fibrosis in phase III trials (160).

The farnesoid X receptor (FXR) is a bile acid-activated nuclear receptor that is abundantly expressed in the liver and intestine (161). Direct activation of FXR enhances anti-inflammatory cytokines (162). Obeticholic acid (OCA), an effective FXR agonist, has been shown to prevent liver fibrosis by inhibiting KCs activation by blocking multiple inflammatory signaling pathways (163). In a Phase III trial, OCA significantly improved fibrosis in patients with MASH (164).

### 7.2 Inhibiting the recruitment of inflammatory cells to the injured liver

The recruitment of inflammatory cells to the injured liver is a critical step in the progression of liver inflammation and fibrosis. This process is largely dependent on the chemotactic effects of various chemokines secreted by activated hepatocytes, macrophages, and HSCs. Among these inflammatory cells, Ly-6C^hi^ MoMϕs are particularly reliant on the signaling pathways involving CCL2/CCR2, CCL1/CCR8, and CCL25/CCR9 (165). Inhibition or elimination of macrophage recruitment via these signaling pathways can significantly ameliorate liver inflammation and global fibrosis in mice. Currently, strategies to interfere with chemokine signal transduction include the use of monoclonal antibodies, receptor antagonists, or small-molecule inhibitors to block chemokine-induced intracellular signaling (8). Among patients with steatohepatitis, the activation of KCs triggers the attraction of BMDMs via the CCR2/CCL2 and CCR5/CCL5 interaction pathways. This process promotes inflammation and contributes to the progression of fibrosis (166).

Notably, a dual inhibitor of CCR2/CCR5, known as Cenicriviroc (CVC), effectively blocks CCL2-mediated monocyte recruitment to the liver and exhibits anti-fibrotic effects in a mouse model of liver fibrosis (167). The phase III clinical trial demonstrated that a 12-month regimen of CVC 150 mg once daily failed to achieve histological improvement in liver fibrosis among MASH patients. However, CVC maintained a favorable safety profile and was well tolerated in this cohort with MASH and liver fibrosis (168).

In addition, medium chain fatty acid receptor G protein junction acceptor 84 (GPR84) has been identified as a mediator of myeloid immune cell infiltration under inflammatory conditions. Small-molecule antagonists (CpdA and CpdB) targeting GPR84 have been shown to obstruct macrophage recruitment to sites of injury in mice with both acute and chronic liver injury, thereby alleviating liver inflammation and fibrosis (169). Moreover, CCR9-deficient HSCs exhibit reduced fibrotic potential *in vitro (* 170). Blocking the CCR9/CCL25 axis with a CCR9 antagonist represents an effective approach to mitigate the progression of hepatic fibrosis (171).

### 7.3 Shaping the heterogeneity of hepatic macrophages

Macrophage phenotypes exert contrasting functions, with pro-inflammatory macrophages typically associated with pro-inflammatory responses and alternatively activated macrophages with anti-inflammatory and tissue-repair functions. Consequently, therapeutic strategies aimed at promoting a switch from a pathogenic phenotype to a restorative phenotype hold promise for accelerating disease resolution and liver regeneration. This can be achieved using therapies that regulate macrophage polarization or reprogram macrophages into a restorative phenotype (155).

β-cryptoxanthin, a lutein carotenoid, has been shown to exert protective effects on markers of hepatic fat accumulation and inflammation (172). β-cryptoxanthin can directly attenuate LPS-induced pro-inflammatory macrophage activation while enhancing IL-4-induced alternatively activated macrophage activation, suggesting that β-cryptoxanthin may represent a promising therapeutic option for patients with liver fibrosis (173). Astaxanthin exhibits stronger antioxidant activity than β-carotene, and is particularly effective in reducing liver inflammation and inhibiting the activation of HSCs (174). It inhibits the activation of JNK/P38 and NF-κB signaling pathways by suppressing T-cell activity, macrophage recruitment, and KCs activation (175). Similarly, astaxanthin has been shown to decrease pro-inflammatory macrophages (176).

Glucagon-like peptide-1 (GLP-1) is a hormone secreted by the gut that lowers blood glucose levels by promoting glucose-dependent insulin secretion and inhibiting glucagon secretion. The glucose-lowering drug liraglutide, an analogue of GLP-1, has shown good efficacy in liver fibrosis (177). *In vitro* experiments showed that liraglutide counteracted the pro-inflammatory polarization of F4/80^+^ macrophages induced by palmitic acid (PA) in wild type mice, mediated through modulation of the cAMP–PKA–STAT3 signaling cascade (178). Moreover, corilagin, a gallotannin, mediating the reprogramming of alternatively activated macrophages to a pro-inflammatory phenotype by regulating the expression of Indoleamine 2,3-dioxygenase 1 (IDO1) *in vitro*, thereby alleviating liver fibrosis (179).

### 7.4 Augmenting the differentiation into restorative macrophages

Peroxisome proliferator-activated receptors (PPARs) play a key regulatory role in the liver, controlling insulin sensitivity, glucose and lipid metabolism, inflammation, and fibrosis (180). PPAR δ plays an anti-inflammatory role by promoting alternatively activated polarization of KCs and decreasing the expression of NLRP3, caspase-1 and IL-1β upon stimulation with saturated fatty acids and LPS. Elafibranor (GFT505), a dual PPARα/δ agonist, has been shown to reduce steatosis, inflammation, and fibrosis in several mouse models of steatohepatitis and decrease the gene expression of pro-inflammatory and pro-fibrotic markers (181). Lanifibranor, as a novel pan-PPAR agonist, decreases the pro-inflammatory activation of macrophages in the liver (182). In a phase 2b trial, it has been indicated that lanifibranor can alleviate liver fibrosis (183).

Gal-3 can directly trigger the NOD-like receptor thermal protein domain associated protein 3 (NLRP3) inflammasome in liver macrophages. Macrophage-derived pro-inflammatory cytokines ultimately result in the cascade of events leading to fibrosis (184). Gal-3 ablation protects mice from diet-induced steatohepatitis and reduces liver inflammation and fibrosis in HFD-fed mice (185). Gal-3 inhibitor GR-MD-02 shows potential efficacy in MASH with advanced fibrosis (186).

GPBAR-1 (TGR5) is a bile acid-activated receptor (BAR) expressed in various liver cells, including KCs, sinusoidal endothelial cells, and HSCs (187, 188). Bar501, a selective ligand of GPBAR-1, can effectively reduce bile duct inflammation, mitigate liver fibrosis and restore bile acid homeostasis (189).

### 7.5 Cell therapy with autologous macrophage infusion

More recently, mesenchymal stem cell (MSC) therapy has emerged as a promising alternative for treating liver diseases (190). MSCs possess the potential to differentiate into hepatocytes and exhibit immunomodulatory properties. They also secrete various trophic factors, including growth factors and cytokines, which have therapeutic implications. In addition, mesenchymal stem cells can inhibit the inflammatory response, reduce hepatocyte apoptosis, promote hepatocyte regeneration, attenuate liver fibrosis, and enhance liver function (191). However, depending on the route of MSC injection and the status of liver disease, MSCs may differentiate into myofibroblasts, thereby exacerbating liver fibrosis. Despite these potential risks, the therapeutic efficacy of MSCs in liver fibrosis has been demonstrated in both preclinical and clinical studies (192).

In addition to MSC therapy, cell therapy involving the transfer of autologous beneficial macrophages has also been explored. Macrophage cell therapy improves clinically relevant parameters in experimental chronic liver injury (193). BMDMs can recruit and modify endogenous macrophages to activate natural killer (NK) cells by modulating the hepatic microenvironment. Pro-inflammatory macrophages also increase the total number of NK cells and activated NK cells in the fibrotic liver, which promoted HSCs apoptosis through TRAIL release. (194).

### 7.6 Targeting macrophages with nanostructures

Nanodrugs have been demonstrated to improve inflammation and liver fibrosis by targeting macrophages. The polarization and reprogramming of macrophages can be differentially modulated by nanoparticles that vary in their physicochemical attributes, such as chemical makeup, size, and surface modification (195). A nanomedicine delivery system has been engineered to target KCs by exploiting receptors that are predominantly present on them, such as mannose and scavenger receptors. This system is intended to deliver a range of therapeutics, including anti-inflammatory medications, ROS scavengers, agents that modify the KCs phenotype, and small interfering RNA (siRNA) drugs aimed at inflammatory mediators, directly to KCs. This approach holds significant promise for the treatment of liver fibrosis (196). A polydatin-loaded micelle demonstrates highly efficient liver-targeted drug release in response to the fibrotic microenvironment (197). Moreover, researchers have developed a dual-drug-loaded lipid nanoparticle. It can effectively suppress macrophage pro-inflammatory signaling and degrade the ECM barrier (198). Therapeutic approaches for targeting macrophages in liver diseases are summarized in **Table 1**.

**Table 1:** Therapeutic approaches for targeting macrophages in liver diseases.

Strategy	Classification and Compound	Mechanism/Cytokines	References
Dampening KC activation	pan-Caspases inhibitor *(Emricasan)*	Inhibiting KCs activation caused by NLRP3 inflammasome cascade and reducing inflammation and apoptosis	(156–158)
	ASK-1 inhibitor *(Selonsertib)*	Inhibiting ASK1 and its downstream P38 and C-Jun N-terminal kinase phosphorylation	(160)
	Bile acid FXR agonist *(Obeticholic acid, GW4064)*	Inhibiting endotoxin-induced KCs activation and liver inflammation	(163, 164)
Inhibiting inflammatory monocyte recruitment to injured liver	CCR2-CCR5 dual antagonist *(Cenicriviroc)*	Blocking CCR2 and CCR5 which mediate inflammatory and fibrotic	(167, 168)
	GPR84 antagonists *(CpdA and CpdB)*	Reducing macrophage accumulation	(169)
Shaping the heterogeneity of Hepatic macrophages	β-cryptoxanthin	Directly decreasing M1 macrophage activation and increasing M2 macrophage activation	(172, 173)
	Astaxanthin	Inhibiting the activation of Jun-N/P38 and NF-κB signaling pathways.	(174, 176)
	GLP-1 analogue (*Liraglutide)*	Regulating the cAMP-PKA-STAT3 signaling. pathway	(177, 178)
Augmenting the differentiation into restorative macrophages	Dual PPARα/δ agonist *(Elafibranor, GFT505)*	Promoting M2 polarization of KCs and decreasing the expression of NLRP3, caspase-1 and IL-1β	(181)
	Pan-PPAR agonist *(Lanifibranor)*	Decreasing pro-inflammatory activation of macrophages	(182, 183)
	Galectin-3 inhibitor *(GR-MD-02, Belapectin)*	Inhibiting a variety of pro-fibrosis factors	(186)
	GPBAR-1 agonist *(Bar501)*	Reducing steatosis, inflammation, and fibrosis	(189)
Cell therapy with autologous macrophage infusion	BMDM	activating NK cells and promoting HSCs apoptosis	(194)
Nanometer carrier	Nanoparticles	Targeting macrophages	(196–198)

## 8 Conclusions and perspectives

In summary, translating the concept of macrophage heterogeneity into clinically effective therapy for liver fibrosis requires addressing two fundamental questions: (i) What are the precise functional roles and pathophysiological significance of distinct macrophage phenotypes across different disease stages? (ii) How can we achieve spatiotemporally precise reprogramming macrophage phenotypes to favor fibrosis resolution while minimizing off-target effects?

The current translational challenges primarily stem from interspecies discrepancies and human-specific complexities. Although murine models have provided foundational insights, they often fail to fully recapitulate the multidimensional heterogeneity of human macrophages, which is shaped by genetic polymorphisms, epigenetic modifications, demographic variables (age, sex, ethnicity), and dynamic host-microbiome interactions. This biological divergence contributes to the frequent discordance between preclinical efficacy and clinical trial outcomes. The functional plasticity of macrophages—acting as double-edged swords in disease initiation (pro-inflammatory), progression (pro-fibrotic), and resolution (pro-reparative)—demands phenotype-specific targeting strategies rather than global macrophage modulation. In addition, macrophage biology in the liver is complicated by phenotypic plasticity, overlapping markers, and inconsistent classification, making it difficult to distinguish between different populations and their functional roles. On the one hand, microenvironmental signals drive rapid phenotypic switching in both KCs and BMDMs, leading to shared surface markers and functional profiles. This bidirectional interconversion blurs the line between resident and recruited macrophages, challenging traditional identification methods. On the other hand, inconsistent nomenclature and unclear definitions further complicate the field. Many studies classify macrophage subsets with distinct names, yet there is significant overlap between datasets, raising questions about whether identified clusters represent true distinct populations or merely different activation states. Additionally, much of the existing research remains descriptive, lacking mechanistic insights into macrophage functions in health and disease.

Future studies would be focused on elucidating the precise roles of distinct macrophage phenotypes at each stage of liver fibrosis and developing targeted therapies that can precisely modulate macrophage function in a spatiotemporal manner. This approach holds promise for improving therapeutic outcomes and addressing the inherent complexities in liver fibrosis.
